# Supplementary material for: The drivers of West Nile virus human illness in the Chicago, Illinois, USA area: Fine scale dynamic effects of weather, mosquito infection, social, and biological conditions
Source: PLoS One. 2020 May 21;15(5):e0227160. doi: 10.1371/journal.pone.0227160 (PMC7241786; doi:10.1371/journal.pone.0227160)
Supplement: S1 Table — (DOCX) [file pone.0227160.s001.docx]

Supplemental Table 1. Candidate models for predicting the probability of human WNV occurrence using weather, land cover, mosquito infection, and demographic factors in Chicago region.

| Model | Variables included | K | -2 log likelihoods | AIC | ΔAIC |
| --- | --- | --- | --- | --- | --- |
| 1 | Yr + templag2- 4 + precilag2 + mirlag1- 4 + whitepct + owpct + dmipct + dhipct | 14 | 12480.5 | 12530.5 | 0 |
| 2 | Yr + templag2- 4 + precilag2 and 4 + mirlag1- 4 + whitepct + owpct + dmipct + dhipct | 15 | 12484.1 | 12536.1 | 5.6 |
| 3 | Yr + templag2- 4 + mirlag1- 4 + whitepct + owpct + dmipct + dhipct | 13 | 12489.3 | 12537.3 | 6.8 |
| 4 | Yr + templag2- 4 + precilag2 + mirlag1- 4 + whitepct + dmipct + dhipct | 13 | 12490.8 | 12538.8 | 8.3 |
| 5 | Yr + templag2- 4 + precilag2 and 4 + mirlag1- 4 + whitepct + income + owpct + dmipct + dhipct | 16 | 12488.7 | 12542.7 | 12.2 |
| 6 | Yr + templag1- 4 + precilag2 and 4 + mirlag1- 4 + income + whitepct + owpct + dmipct + dhipct | 17 | 12503.5 | 12559.5 | 29 |
| 7 | Yr + templag1- 4 + precilag1-2 and 4 + mirlag1- 4 + income + whitepct + owpct + dmipct + dhipct | 18 | 12502.6 | 12560.6 | 30.1 |
| 8 | Yr + templag1- 4 + precilag1- 4 + mirlag1- 4 + income + whitepct + owpct + dmipct + dhipct + mfpct + glandpct + wwpct | 22 | 12502.6 | 12560.6 | 30.1 |
| 9 | Global model (all predictor variable included) | 33 | 12476.47 | 12566.5 | 36 |
| 10 | Null model | 1 | 14210.7 | 14214.7 | 1684.2 |
