# Supplementary material for: The drivers of West Nile virus human illness in the Chicago, Illinois, USA area: Fine scale dynamic effects of weather, mosquito infection, social, and biological conditions
Source: PLoS One. 2020 May 21;15(5):e0227160. doi: 10.1371/journal.pone.0227160 (PMC7241786; doi:10.1371/journal.pone.0227160)
Supplement: Attachment — Submitted filename: Response to Editor.docx [file pone.0227160.s004.docx]

Response to Editor:

In addition, please elaborate more to render Figure captions self explained by including all respective information.

Also, the references list should be checked carefully and editing mistakes should be corrected. For example all scientific names at species and genus level should be given in italics.

Corrected

Last table 5 could be included as supplementary materials.

Done

Please consider the attached annotated pdf and also make some additional proofreading especially in reference.

All corrections have been made
